# Epidemiology of *Neisseria gonorrhoeae* Gyrase A Genotype, Los Angeles, California, USA

**DOI:** 10.3201/eid2309.170215

**Published:** 2017-09

**Authors:** Ashima A. Bhatti, Lao-Tzu Allan-Blitz, Mariana Castrejon, Romney M. Humphries, Peera Hemarajata, Jeffrey D. Klausner

**Affiliations:** University of California, Los Angeles, California, USA (A.A. Bhatti, L.-T. Allan-Blitz, M. Castrejon, R.M. Humphries, P. Hemarajata, J.D. Klausner);; UCLA Health, Los Angeles (M. Castrejon, J.D. Klausner)

**Keywords:** Neisseria gonorrhoeae, bacteria, gyrase A, gyrase A gene, gyrA, genotype, epidemiology, sexually transmitted infections, men who have sex with men, antimicrobial resistance, ciprofloxacin, gonorrhea, Los Angeles, California, USA

## Abstract

We investigated the epidemiology of the mutant gyrase A gene, a reliable predictor of ciprofloxacin resistance, in *Neisseria gonorrhoeae* infections at UCLA Health in Los Angeles, California, USA, during November 1, 2015–August 31, 2016. Among 110 patients with *N. gonorrhoeae* infections, 48 (44%) had the mutant gyrase A gene.

In 2013, the Centers for Disease Control and Prevention (Atlanta, GA, USA) declared that multidrug-resistant *Neisseria gonorrhoeae* infections were 1 of the top 3 urgent antimicrobial drug resistance threats (*1*). To combat this growing threat, we developed and implemented a real-time reverse transcription PCR at the University of California, Los Angeles (Los Angeles, CA, USA), to detect the codon 91 mutation in the gyrase A (*gyr*A) gene in *N. gonorrhoeae* remnant clinical specimens (*2*). Mutations in the *gyr*A gene of *N. gonorrhoeae*, specifically at codon 91, have been demonstrated to reliably predict resistance to ciprofloxacin (*3*).

Current clinical practice uses mostly nucleic acid amplification tests to detect *N. gonorrhoeae* and not culture-based methods (*4*). Although those tests yield better clinical outcomes, they do not provide useful antimicrobial drug susceptibility data. Therefore, factors associated with antimicrobial drug resistance in the general community are not routinely available (*4**,**5*).

## The Study

We characterized the epidemiology of fluoroquinolone resistance at UCLA Health (Los Angeles, CA, USA) by using a rapid molecular *gyr*A gene assay that predicts ciprofloxacin susceptibility (*3**,**6*). UCLA Health is a large healthcare system in Los Angeles County compound of 2 hospitals, 2 emergency departments, and >150 primary care clinics serving ≈500,000 patient-visits each year. We reviewed electronic patient medical records for November 1, 2015–August 31, 2016, for all cases of *N. gonorrhoeae* infection detected by using the Cobas 4800 CT/NG Assay (Roche Molecular Systems, Pleasanton, CA, USA).

We collected data on age, sex, sex of sex partners, race/ethnicity, HIV infection status, pregnancy, HIV preexposure prophylaxis use, *gyrA* gene results by anatomic site of infection, presence of other sexually transmitted infections, substance use, history of fluoroquinolone exposure in the past 2 years, and history of previous *N. gonorrhoeae* infection. Each positive *N. gonorrhoeae* result in a single patient was considered a unique infection. The date of *N. gonorrhoeae*–specimen collection was considered the infection date.

Patients were considered to have no fluoroquinolone exposure only if they had medical records going back >2 years from infection date and no documentation of having been prescribed a fluoroquinolone during that time. Patients were considered to have no history of previous *N. gonorrhoeae* infection if they had medical records going back >2 years from current infection date and no documentation of having a positive *N. gonorrhoeae* test result at UCLA Health during those 2 years.

Descriptive statistics, prevalence ratios (PRs), and p values by χ^2^ test or Fisher exact test are reported. We performed analysis by using STATA software version 14.2 (StataCorp LLC, College Station, TX, USA). UCLA determined that analysis of unidentified data was exempt from ethical review.

Among 141 patients for whom *N. gonorrhoeae* genotyping was attempted, 110 (78%) had a genotype identified; 31 (22%) had an indeterminate genotype. Of the 110 patients who had a genotype identified, 48 (44%) had a mutant genotype and 62 (56%) had a wild-type genotype. Twenty (18%) of the patients were women, 1 of whom was a transgender woman. The remaining 90 (82%) patients were men, and 58 (64%) were men who have sex with men (MSM). Forty (42%) patients who had documentation of HIV status were infected. Sixteen women had pregnancy tests performed; 6 (37%) of these women were pregnant. We obtained demographic characteristics and other factors for all patients (Table).

**Table:** Characteristics of 110 patients infected with *Neisseria gonorrhoeae* containing gyrase A mutant and wild-type genes, UCLA Health, Los Angles, California, USA, November 1, 2015–August 31, 2016*

Characteristic	No. (%)	No. (%) with *gyrA *mutant gene	Prevalence ratio (95% CI)	p value†
No. patients	110	48 (44)		
Age, y, n = 110				
17–34	68 (62)	29 (43)	1	Referent
>35	42 (38)	19 (45)	1.1 (0.69–1.6)	0.79
Sex and sexual orientation, n = 110				
Men who have sex with men	58 (53)	25 (43)	1	Ref
Men who have sex with women only	11 (10)	6 (55)	1.3 (0.68–2.3)	0.48
Men of unknown orientation	21 (19)	10 (48)	1.1 (0.65–1.9)	0.72
Women	20 (18)	7 (35)	0.81 (0.42–1.6)	0.52
Race/ethnicity, n = 110				
White	42 (38)	16 (38)	1	Referent
Hispanic	12 (11)	3 (25)	0.66 (0.23–1.9)	0.51‡
Black/African American	20 (18)	12 (60)	1.6 (0.93– 2.7)	0.11
Asian or Indian	5 (5)	2 (40)	1.1 (0.37–6.8)	1‡
Other or nonspecified	31 (28)	15 (48)	1.3 (0.75–2.2)	0.38
HIV infection status, n = 95				
Uninfected	55 (58)	24 (44)	1	Referent
Infected	40 (42)	18 (45)	1.03 (0.65–1.6)	0.89
Genotype by anatomic site, n = 125 samples				
Pharyngeal	19 (15)	5 (26)	1	Referent
Cervical/vaginal	7 (5.6)	3 (43)	1.6 (0.52–5.1)	0.64‡
Rectal	35 (28)	17 (49)	1.8 (0.81–4.2)	0.11
Urethral	64 (51)	27 (42)	1.6 (0.72–3.6)	0.21
History of *N. gonorrhoeae* infection, n = 77				
No	55 (71)	21 (38)	1	Referent
Yes	22 (29)	12 (55)	1.4 (0.86–2.4)	0.19
Other sexually transmitted infections, n = 110				
None	71 (65)	33 (46)	1	Referent
* Chlamydia trachomatis*	31 (28)	13 (42)	0.90 (0.56–1.5)	0.67
* Treponema pallidum*	6 (5)	1 (17)	0.36 (0.06–2.2)	0.22‡
* Trichomonas vaginalis*	2 (2)	1 (50)	1.1 (0.26–4.4)	1‡
Pregnant, n = 16				
No	10 (63)	3 (30)	1	Referent
Yes	6 (37)	2 (33)	1.1 (0.25–4.9)	1‡
Recent methamphetamine or heroin use, n = 66				
No	59 (89)	30 (51)	1	Referent
Yes	7 (11)	3 (43)	0.84 (0.35–2.1)	1‡
PrEP use, n = 73				
No	56 (77)	22 (39)	1	Referent
Yes	17 (23)	8 (47)	1.2 (0.66–2.2)	0.57
History of fluoroquinolone exposure, n = 76				
No	61 (80)	26 (43)	1	Referent
Yes	15 (20)	9 (60)	1.4 (0.85–2.3)	0.23
Past 3 mo	2 (3)	2 (100)	2.3 (1.8–3.1)	0.19‡
Past 4–12 mo	9 (12)	6 (67)	1.6 (0.91–2.7)	0.28‡
Past 13–24 mo	4 (5)	1 (25)	0.59 (0.10–3.3)	0.64‡

*n values indicate number of patients for which data were available or total number of samples collected. *gyrA*, gyrase A; PrEP, preexposure prophylaxis. †By χ^2^ test unless otherwise noted. ‡By 2-sided Fisher exact test.

Among 35 patients with a *gyrA* mutant genotype who had medical records going back >2 years, 9 (26%) were exposed to fluoroquinolones during that time, compared with 6 (15%) of 41 patients with wild-type *gyrA* genotype with medical records going back >2 years (PR 1.4, 95% CI 0.85–2.3; p = 0.23). A recent study demonstrated that treatment might be a major driver of resistance (*7*), and a previous study demonstrated that a history of fluoroquinolone exposure is associated with an increased prevalence of fluoroquinolone resistance (*8*). The lack of statistical significance between previous fluoroquinolone exposure and presence of *N. gonorrhoeae* mutant *gyrA* genotype in our study might be caused by small sample size.

Patients >35 years of age were more likely to have medical records going back >2 years than persons 17–34 years of age (PR 1.6, 95% CI 1.2–2.0; p<0.001). Men were more likely than women to have medical records going back >2 years (PR 1.6, 95% CI 1.0–2.5; p = 0.048). MSM were more likely to have medical records going back >2 years than all other men (PR 1.4, 95% CI 1.06–1.9; p = 0.019). These findings indicate the possibility of selection bias regarding medication exposure in our study.

During the 10-month study period, there were 171 site-specific *N. gonorrhoeae* infections. For these infections, none of the patients with infections at multiple anatomic sites showed discordant genotypes. One patient with repeat infections during the study showed a change in *gyrA* genotype from wild-type to mutant during the subsequent infection. Most infections were successfully genotyped regardless of anatomic site, except for the pharyngeal site, which yielded genotypes for only 37% of infections (Figure, panel A). There were no differences in distribution of genotype among anatomic sites (Figure, panel A; Table).

**Figure:**
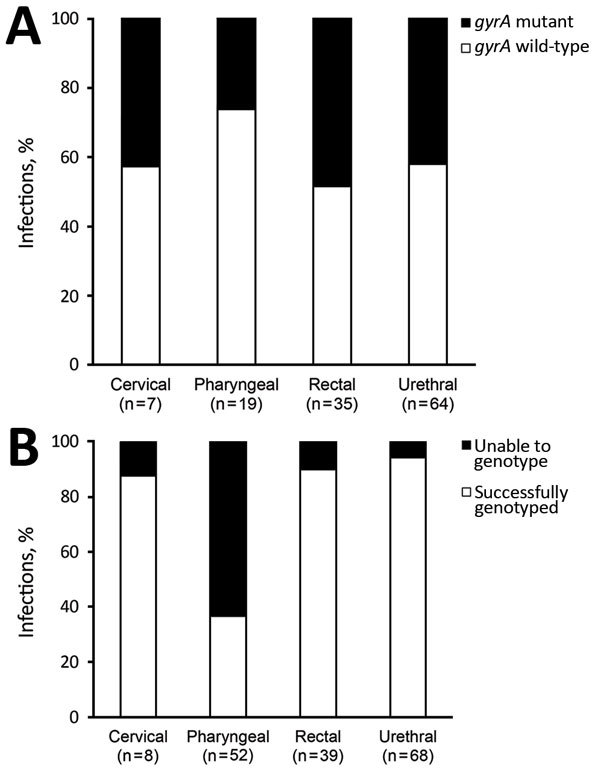
Proportion of *Neisseria gonorrhoeae* infections genotyped for gyrase A gene by anatomic site, UCLA Health, Los Angeles, California, USA, November 1, 2015–August 31, 2016. A) Gyrase A gene; B) gyrase A mutant and wild-type genes.

Of the 42 infections that could not be genotyped, 32 were in 31 patients who had no other genotyped infections. These infections were excluded from our analyses. The remaining 10 infections were in patients who had a genotyped infection at another anatomic site: 9 of these patients were MSM and 1 was a woman. There was no major difference in distribution of sexual orientation, age, or genotype for indeterminate samples with identified genotypes for another simultaneous infection compared with those that did not have successful genotyping of another simultaneous infection.

## Conclusions

Previous studies have reported a similar low sensitivity of the *N. gonorrhoeae gyrA* gene assay for pharyngeal specimens, but the cause remains uncertain (*6**,**9*). It was previously believed that PCR inhibitors were present in pharyngeal reservoirs, perhaps from commensal *Neisseria* spp.; however, this suggestion is no longer believed to be the case (*6**,**9*).

Our study had some limitations. First, patients who had medical records that go back <2 years might have received fluoroquinolones or had *N. gonorrhoeae* infections diagnosed at other institutions. If we were able to access that information, we would probably see a stronger association between mutant *gyrA* genotype and fluoroquinolone exposure and history of *N. gonorrhoeae* infection. Second, the UCLA Health system is a large, well-established institution in a large metropolitan area. Therefore, our results might not be representative of trends in *N. gonorrhoeae* resistance in other regions or healthcare systems.

In summary, we found a prevalence of 44% for the mutant *gyrA* genotype, which confers ciprofloxacin resistance, among patients infected with *N. gonorrhoeae*. Molecular methods to predict susceptibility testing offer a potential new way to monitor *N. gonorrhoeae* drug resistance in the United States. Replication of our work in other settings is urgently needed.

## References

[1] Barton J, Braxton J, Darlene D, de Voux A, Flagg E, Grier L, et al. Division of STD Prevention, Centers for Disease Control and Prevention. Sexually transmitted disease surveillance 2015. Atlanta. October 2016 [cited 2017 Jun 12]. https://www.cdc.gov/std/stats15/std-surveillance-2015-print.pdf

[2] Allan-Blitz LT, Humphries RM, Hemarajata P, Bhatti A, Pandori MW, Siedner MJ, et al. Implementation of a rapid genotypic assay to promote targeted ciprofloxacin therapy of *Neisseria gonorrhoeae* in a large health system. Clin Infect Dis. 2017;64:1268–70. 28034887 10.1093/cid/ciw864 PMC5399946

[3] Allan-Blitz LT, Wang X, Klausner JD. Wild-type gyrase A genotype of *Neisseria gonorrhoeae* predicts in vitro susceptibility to ciprofloxacin: a systematic review of the literature and meta-analysis. Sex Transm Dis. 2017;44:261–5. 10.1097/OLQ.0000000000000591 28407640 PMC5407314

[4] Garrett TA, Davies-Cole J, Furness B. Laboratory capacity for antimicrobial susceptibility surveillance of *Neisseria gonorrhoeae*, District of Columbia, 2007–2012. Sex Transm Dis. 2015;42:413–6. 10.1097/OLQ.0000000000000304 26165430 PMC7140763

[5] Hook EW III. Generalized testing for gonococcal antibiotic susceptibility or sentinel surveillance. Sex Transm Dis. 2015;42:417–8. 10.1097/OLQ.0000000000000321 26165431

[6] Hemarajata P, Yang S, Soge OO, Humphries RM, Klausner JD. Performance and verification of a real-time PCR assay targeting the *gyrA* gene for prediction of ciprofloxacin resistance in *Neisseria gonorrhoeae.* J Clin Microbiol. 2016;54:805–8. 10.1128/JCM.03032-15 26739156 PMC4767994

[7] Fingerhuth SM, Bonhoeffer S, Low N, Althaus CL. Antibiotic-resistant *Neisseria gonorrhoeae* spread faster with more treatment, not more sexual partners. PLoS Pathog. 2016;12:e1005611. 10.1371/journal.ppat.1005611 27196299 PMC4872991

[8] Klausner JD, Aplasca MR, Mesola VP, Bolan G, Whittington WL, Holmes KK. Correlates of gonococcal infection and of antimicrobial-resistant *Neisseria gonorrhoeae* among female sex workers, Republic of the Philippines, 1996-1997. J Infect Dis. 1999;179:729–33. 10.1086/314625 9952388

[9] Low N, Unemo M. Molecular tests for the detection of antimicrobial resistant *Neisseria gonorrhoeae*: when, where, and how to use? Curr Opin Infect Dis. 2016;29:45–51. 10.1097/QCO.0000000000000230 26658656

